# Membrane-Based Electrolysis for Hydrogen Production: A Review

**DOI:** 10.3390/membranes11110810

**Published:** 2021-10-24

**Authors:** Mohd Fadhzir Ahmad Kamaroddin, Nordin Sabli, Tuan Amran Tuan Abdullah, Shamsul Izhar Siajam, Luqman Chuah Abdullah, Aishah Abdul Jalil, Arshad Ahmad

**Affiliations:** 1 Department of Chemical and Environmental Engineering, Faculty of Engineering, Universiti Putra Malaysia, Serdang 43400 UPM, Selangor, Malaysia; shamizhar@upm.edu.my (S.I.S.); chuah@upm.edu.my (L.C.A.); 2 Centre of Hydrogen Energy, Institute of Future Energy, Universiti Teknologi Malaysia, Skudai 81310 UTM, Johor, Malaysia; tuanamran@utm.my (T.A.T.A.); aishahaj@utm.my (A.A.J.); arshad@utm.my (A.A.); 3 Institute of Advanced Material, Universiti Putra Malaysia, Serdang 43400 UPM, Selangor, Malaysia; 4 School of Chemical and Energy Engineering, Faculty of Engineering, Universiti Teknologi Malaysia, Skudai 81310 UTM, Johor, Malaysia

**Keywords:** membrane, electrolysis, hydrogen production, electrolysis technologies, zero-carbon footprint, water splitting technologies, membrane-based electrolysis, electrolyzer, efficient

## Abstract

Hydrogen is a zero-carbon footprint energy source with high energy density that could be the basis of future energy systems. Membrane-based water electrolysis is one means by which to produce high-purity and sustainable hydrogen. It is important that the scientific community focus on developing electrolytic hydrogen systems which match available energy sources. In this review, various types of water splitting technologies, and membrane selection for electrolyzers, are discussed. We highlight the basic principles, recent studies, and achievements in membrane-based electrolysis for hydrogen production. Previously, the Nafion™ membrane was the gold standard for PEM electrolyzers, but today, cheaper and more effective membranes are favored. In this paper, CuCl–HCl electrolysis and its operating parameters are summarized. Additionally, a summary is presented of hydrogen production by water splitting, including a discussion of the advantages, disadvantages, and efficiencies of the relevant technologies. Nonetheless, the development of cost-effective and efficient hydrogen production technologies requires a significant amount of study, especially in terms of optimizing the operation parameters affecting the hydrogen output. Therefore, herein we address the challenges, prospects, and future trends in this field of research, and make critical suggestions regarding the implementation of comprehensive membrane-based electrolytic systems.

## 1. Introduction

The world’s population as of August 2021 is almost 7.9 billion, as reported by the United Nations, surpassing the earlier prediction of 7.5 billion by 2025 [1]. The world needs enough food for the entire population. To fulfill this need, energy resources are required to move people around, powering agriculture and agro-based industries, as well as other activities [2,3,4]. It is anticipated that the world’s energy demand will be in the range of 600 to 1000 EJ by 2050 [5,6,7]. A smart approach is essential to balance power demands and effective management of produced energy [8,9]. Due to the intense usage of conventional fuels in the production of electricity, the depletion of ozone layer is now at an alarming level because of the effect of the greenhouse gas (GHG) emissions like carbon dioxide and methane [10,11,12]. As the world is united and committed to reducing GHG emissions, the Montreal Protocol (1987), Kyoto Protocol (1997) and Paris Agreement (2015) have been signed in the hope of preventing further damage to the ozone layer and reducing the impact of climate change by 2050 [13,14,15,16]. Unlike the Montreal and Kyoto protocols (that targeted only developed nations), the Paris Agreement (2015) is more universal in its ambition to reduce GHG emissions, setting a target of a maximum of a 2 °C temperature increase through the collective commitment of all nations to cut their pollution levels [17,18].

**Figure 1:**
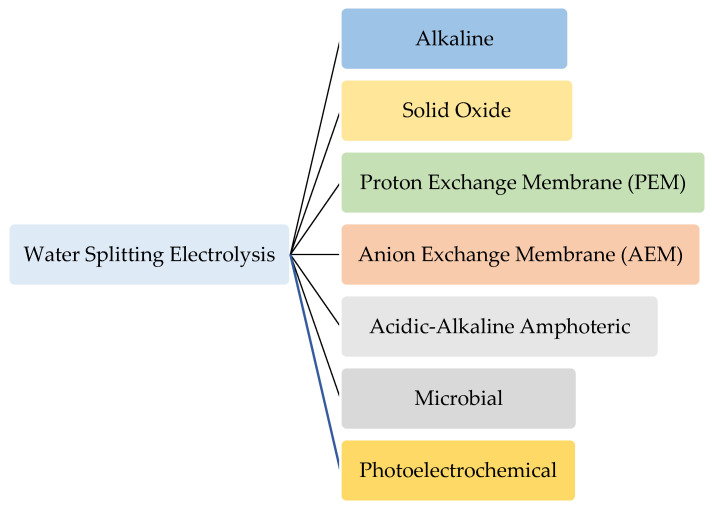
Water splitting electrolysis technologies for hydrogen production.

One of the most promising clean and green energy sources, i.e., without any GHG emission and with a zero carbon footprint, is green hydrogen [19,20]. Hydrogen does not occur naturally in a gas form; rather, it always occurs as a compound in compounds such as water (H_2_O), methane (CH_4_), butane (CH_4_H_10_), or other liquids and hydrocarbon gases [21,22]. There are many techniques to produce hydrogen. For example, it can be produced from renewable sources as in the biomass [23] and water splitting processes (thermolysis, photolysis, electrolysis) [18,24]. Electrolysis can be further divided into alkaline, solid oxide, PEM, AEM, acidic–alkaline amphoteric, microbial and photoelectrochemical, as depicted in Figure 1.

At present, nonrenewable, fossil fuel-based processes, specifically, steam reforming of methane, coal gasification and other chemical processes, account for 96% of worldwide hydrogen generation, with electrolysis contributing just 4% [25,26,27,28]. Nonetheless, hydrogen originating from fossil fuels is low in purity and leads to the release of greenhouse gases including carbon monoxide, sulfur oxides, nitrogen oxides and carbon dioxide [19,29,30]. Hydrogen has several appealing features as an energy vector, including a high heating value (140 MJ/kg) that is almost three times that of conventional petroleum fuels (50 MJ/kg) [9,19]. Currently, global hydrogen production is estimated to be approximately 500 billion cubic meters per year. Hydrogen is widely utilized in a variety of sectors, including in the production of fertilizers, petrochemical processes, energy generation from fuel cells, and in various chemical industries [20,23,31].

There is also a need for innovative energy techniques with zero carbon footprint because of ever-increasing global energy demands and the limited supply of fossil fuels [7,23,32]. Environmentally friendly energy solutions are gaining traction today as viable alternatives to fossil fuel-based systems. It is estimated that less than 1% of the world’s hydrogen consumption is met by green hydrogen, i.e., which used renewable sources in its production [9,33]. One ecological method is polymer electrolyte water electrolysis; this approach yields hydrogen with a purity of hydrogen up to 99.999% [33,34,35].

Green hydrogen is produced from 100% renewable sources in an electrolysis process that uses fully renewable power and generates pure oxygen and hydrogen [25,35]. Gray hydrogen refers to the hydrogen synthesized via the steam methane reforming (SMR) method, as well as the residual hydrogen from chemical processes in chlor-alkali plants. Blue hydrogen refers to a gray hydrogen that has undergone a postproduction step called carbon capture and utilization (CCU) process [25,35,36]. 

The term “hydrogen economy” was introduced by John Bockris at the General Motors Technical Centre in 1970, in reference to a potential future method of generating energy [37]. Today, in Malaysia, the same term refers to the distribution of energy derived from hydrogen rather than fossil-fuel-based systems [38]. Over the course of the half century since the term was coined, the hydrogen technology landscape has evolved tremendously. Hydrogen is seen by some as the ultimate solution to climate change [14,25]. This is because, by introducing green hydrogen production, there will be a zero carbon footprint [24,39].

Although there are other methods of producing hydrogen, the advantages of membrane-based electrolysis include no net carbon release into the atmosphere (only hydrogen, oxygen and water are generated during the operation), ease of replication and the ability to combine multiple single unit membrane electrodes into a stack. Most of all, membrane-based electrolysis can be customized according to specific needs, location, and resource availability [40,41]. Therefore, this paper will review current membrane-based electrolysis for hydrogen production technologies. 

## 2. Types of Membranes for Hydrogen Production

Membranes come in a solid polymer exchange strip that separates the two electrodes, acts as an ion conductor and prevents any fuel diffusivity [42,43,44,45]. In a membrane-based electrolysis process, a good quality membrane is vital to ensure durable operation and sufficient purity of the output product. Perfluorinated sulfonic acid (PFSA) type membranes are now the most frequently utilized solid electrolytes for proton exchange membrane fuel cell (PEMFCs) and proton exchange membrane electrolyzers (PEMEs) [46,47,48,49]. The phase inversion method is the most frequently used method for the production of polymeric membranes [50,51]. Below are some of the characteristics of a high-performance membrane for hydrogen production [21,52,53]:

- High thermal and mechanical stability
- Cost-effective and economic fabrication process
- Excellent ionic conductivity
- Excellent electrical insulation
- High oxidative and hydrolytic stability
- Excellent ability to block ion crossover via membrane/low diffusivity
- Low swelling
- Easy fabrication of the membrane electrode assemblies (MEA)
- High chemical/electrochemical stability

### 2.1. Nafion™

**Figure 2:**
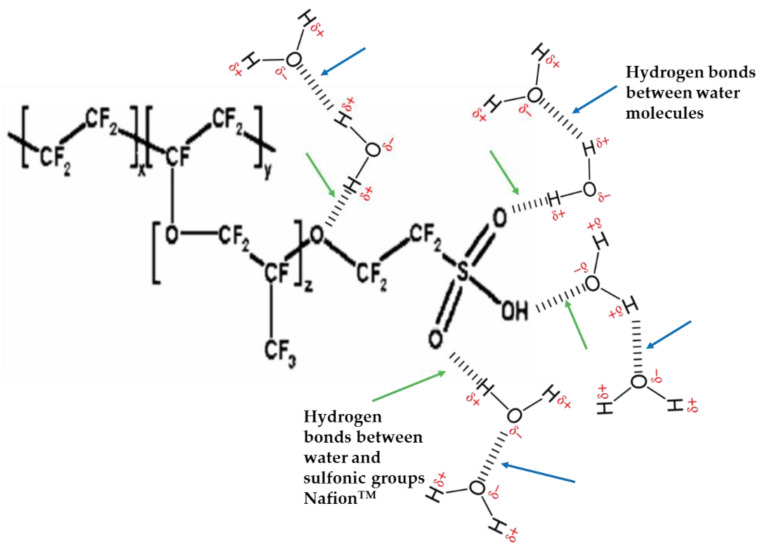
Nafion™ chemical structure with hydrogen bonds (sulfonic group with water molecules) and hydrogen bonds (two water molecules). Modified from [55].

Nafion™ is a well-known perfluorosulfonic acid (PFSA) membrane that is frequently utilized in PEM fuel cells and PEM electrolyzers [21,52,54]. Nafion™ functions well and is currently very popular because of its good ionic conductivity and excellent physicochemical properties. In 1966, General Electric Co. (Boston, MA, USA) was the first to create a proton exchange membrane (PEM) electrolyzer from a solid polymer electrolyte; it consisted of a membrane, an anode, and a cathode. DuPont’s Nafion™ membrane is the most well-known membrane; it is composed of a perluorinated polymer with sulfonic acid functionalization, as shown in Figure 2.

The significance of Nafion™ in the field of fuel cells and electrolyzers is apparent. It has excellent mechanical strength, proton conductivity, and chemical and thermal stability [56,57,58]. However, its apparent flaw, which has yet to be resolved, is the high fuel permeability, which causes PEM fuel cell and direct methanol fuel cell systems to lose a lot of fuel, reducing performance [42,52,59]. There are also ion crossovers in PEM electrolyzers which decrease the hydrogen yield. Furthermore, Nafion™ is very expensive due to the high production cost of the membrane [60,61].

Recently, many attempts have been made to address these shortcomings, including the introduction of inorganic fillers, acid doping and the introduction different polymer backbones into the Nafion™ membrane [27,62]. Moreover, operating an electrolyzer cell at higher temperatures induces more efficient hydrogen production due to the increase in ionic conductivity and a reduction in the anode and cathode activation overpotential [63]. In PEM fuel cells, operating at higher temperatures improves performance by decreasing carbon monoxide (CO) emissions; however, it also accelerates the degradation of the fuel cell components [64]. In the electrolyzer, a higher operating temperature results in an increased hydrogen yield [65].

### 2.2. Polybenzimidazole (PBI)

**Figure 3:**
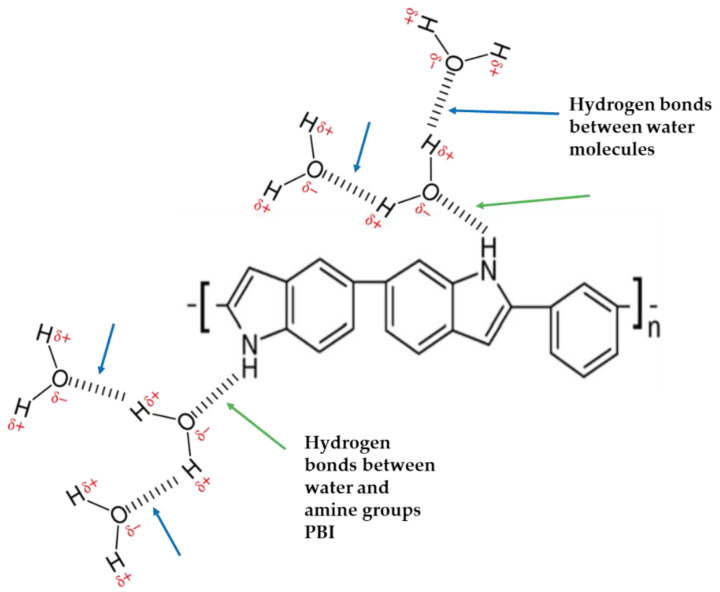
PBI structure with hydrogen bonds (an amine group and water molecules) and hydrogen bonds (two water molecules). Modified from [66].

Polybenzimidazole (PBI) is a term denoting the presence of several benzimidazole units in the structure of aromatic heterocyclic polymers. Compared to Nafion™ membranes, PBI offers a few benefits, including high tensile strength, good chemical stability, and exclusive affinity for polyaryletherketone and some other polymers. The production of PBI is depicted in Figure 3.

The rigid aromatic structure in polybenzimidazole (PBI) contributes to its good chemical stability, high mechanical strength, and remarkable thermal stability. Owing to these characteristics, polybenzimidazole-based (PBI-based) membranes have been intensively explored for use in fuel cells, water electrolysis, and flow batteries [67,68,69].

Even though Nafion™ membranes are excellent at operating temperatures ranging from 20 to 80 °C, they are not suitable for high-temperature applications (>100 °C) due to their mechanical instability and the considerable drop in proton conductivity that occurs with elevated temperature [21,44,48]. Wainright first used polybenzimidazole (PBI) for high-temperature polymer electrolyte membranes in 1995 [27,64]. However, compared to Nafion™, pure PBI has relatively low conductivity, making it unsuitable as a substitute. 

The proton conductivity of pure PBI can be improved by treating it with a variety of inorganic acids via hybrid membrane synthesis methods. For example, ion cross-linked structures can be prepared by blending PBI with sulfonated polyether ether ketone (SPEEK), sulfonated polysulfone, or sulfonated partially fluorinated arylene polyether [30,70]. The proton conductivity of phosphoric acid (PA) -doped PBI membranes is significantly dependent on the acid doping level, which is defined as the number of PA molecules per polymer repeating unit. The proton conductivities of acid-doped PBI membranes are also influenced by the doping acids in the following order: H_2_SO_4_ > H_3_PO_4_ > HClO_4_ > HNO_3_ > HCl. Due to the presence of more effective acid sites, sulfonated PBI membranes have greater proton conductivity than pure PBI membranes [71]. 

### 2.3. Sulfonated Polyether Ether Ketone (SPEEK)

**Figure 4:**
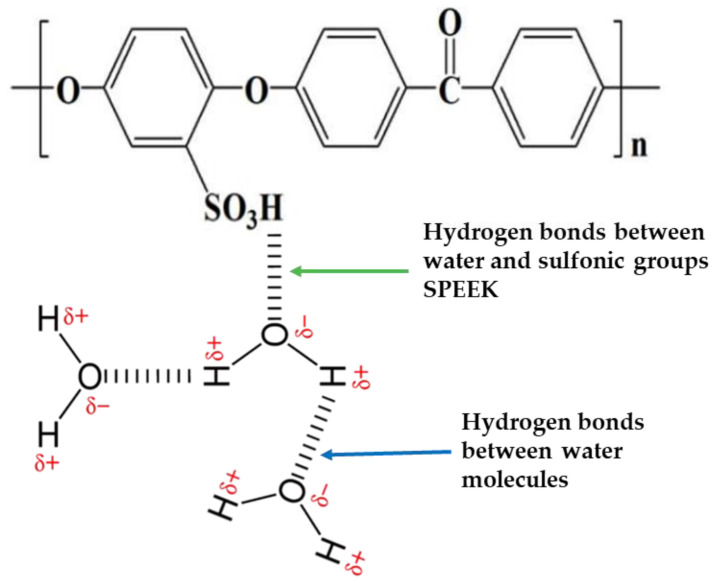
SPEEK chemical structure with hydrogen bonds (sulfonic group and water molecules) and hydrogen bonds (two water molecules). Modified from [75].

Victrex is now the world’s top producer of PEEK polymers. In the sulfonation procedure for SPEEK membranes (Figure 4), sulfonic acid groups (SO_3_H) are attached, via alteration or polymerization of sulfonated monomers, to the backbone structure of the PEEK polymer [72]. The excess acid in the form of sulfonic acid groups in the PEEK polymer is the basis for the hydrophilic properties of the membrane. The sulfonic acid groups serve as hydrogen bonding sites between the polymer and the water [52,70]. Proton charge carriers are formed in PEEK hydrated membranes as a result of sulfonic acid group segregation and proton conductivity due to water activity [73,74].

Previous research has demonstrated that PEEK polymers with altered characteristics can be used to replace Nafion™ membranes in PEMFC, DEMFC, and PEME systems [27,50]. PEEK electrophilic sulfonation (S-PEEK), SPEEK and nonfunctional polymer mixing, and SPEEK heteropolycompounds with poly-etherimide doping with organic acids are all required in order to PEM from PEEK polymer [50,73]. Therefore, controlling the degree of sulfonation (DS) is critical, since this affects the thermochemical stability of PEEK-based membranes [76,77].

### 2.4. Others

Apart from Nafion™, PBI, and SPEEK, other base membranes could be used in hydrogen production processes. For example, other polymers with aromatic rings, such as polyoxadiazole, polysulfone (PSf), and polyimides, could reduce production costs while providing adequate physicochemical properties; however, these compounds need further improvements and investigation. Additionally, the physicochemical properties of these membranes could further be improved by hybrid membrane preparation (solution mixing, acid doping etc). Currently, these polymers and their derivatives (e.g., polyimides/SPAES, polysulfone/PEEK) are mainly used in fuel cell applications and, occasionally, in water electrolysis [31,78].

## 3. Types of Water Electrolysis Technologies

Electrolysis technologies have existed for more than 100 years. At present, fuel cells (which use the opposite process to electrolyzers) are more popular than traditional hydrogen conversion tools for applications in the automobile industry. In the hydrogen production process, the electrolyzer is the most important component, as it determines the production efficiency [79]. 

### 3.1. Nonmembrane-Based Electrolysis

#### Alkaline Electrolysis

**Figure 5:**
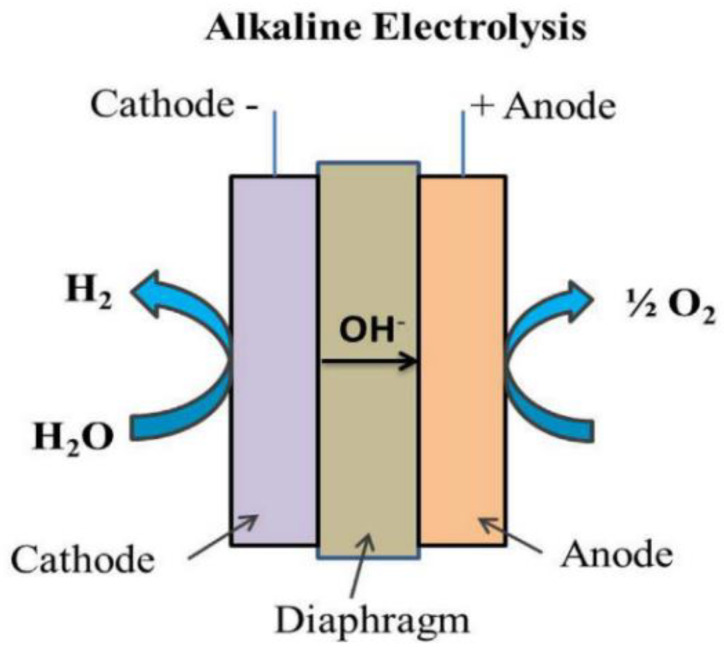
Alkaline electrolysis schematic. Reproduced with permission from [19].

Hydrogen production by electrolysis of alkaline water is now a mature technology that is economical, durable, and has been widely used in chlor-alkali chemical industries for more than 100 years [79,80]. The drawbacks of having an alkaline electrolysis system are low hydrogen purity, limited current density (below 400 mA/cm^2^), low range of operating pressure with low energy efficiency [81,82,83]. The schematic for alkaline electrolysis is shown in Figure 5. 

The half-cell reaction at the anode in an alkaline electrolysis is shown in Equation (1):

Anode: 2OH^−^ → H_2_O + ½ O_2_ + 2e^−^ (1)

As for the cathode, the half-cell reaction in alkaline electrolysis is depicted in Equation (2).

Cathode: 2H_2_O + 2e^−^ → H_2_O + 2OH^−^ (2)

The overall reaction for alkaline electrolysis is represented by Equation (3):

Overall: H_2_O → H_2_ + ½ O_2_ (3)

The hydrogen evolution reaction (HER) starts when the water molecule is reduced at the cathode, producing one hydrogen (H_2_) molecule and two hydroxyl ions (OH^-^) [34,84]. The hydroxyl ions then move to the anode via the porous diaphragm due to the electrical potential which is applied at both electrodes, releasing half a molecule of oxygen (O_2_) and one molecule of water (H_2_O) [82]. Typically, alkaline electrolyzers use 30 wt% KOH solution or 25 wt% NaOH solution and operate at 30–80 °C. These devices are able to produce hydrogen which is up to 99% pure with an efficiency of around 60–80% [79,83]. 

### 3.2. Membrane-Based Electrolysis

#### 3.2.1. Proton Exchange Membrane Electrolysis

Proton exchange membranes (PEMs) are widely used in fuel cells to produce electricity and in electrolyzers to produce hydrogen. PEMs also act as a means of separating the anode from the cathode. Nafion™ and Nafion™-based membranes are the most popular PEMs due to their high ionic conductivity, thermostability, good mechanical strength, excellent chemical stability, and durability at low temperature under high levels of relative humidity [56,84,85,86]. However, Nafion™ has two major problems, i.e., a time-consuming synthesis procedure and poor proton conductivity at high-temperatures in low humidity environments [62,87]. Moreover, the main obstacles for the use of Nafion™ membranes are their exorbitant price, the unsafe membrane synthesis process, and the fact that ionic conductivity drops when the operating temperature exceeds 90 °C under low relative humidity [49,88,89]. 

**Figure 6:**
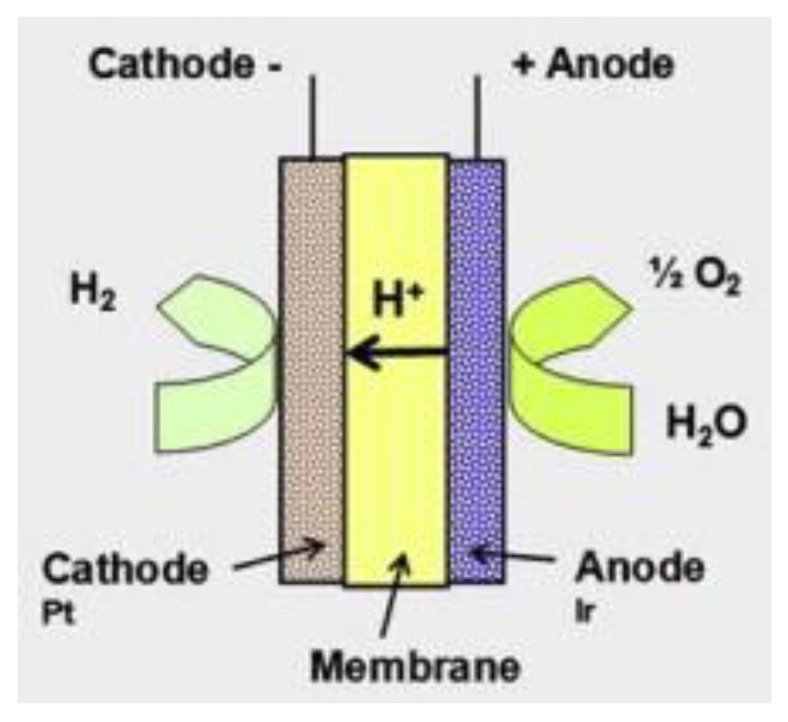
PEM electrolysis. Reproduced with permission from [30].

The advantages of PEM electrolyzers are their abilities to operate at high current densities with high voltage and to produce a very pure hydrogen gas, i.e., up to 99.995% [90]. The downsides of using a PEM electrolysis system are the high cost of the catalyst and the need for an expensive membrane which has only average durability. Furthermore, PEM electrolyzer stack materials are more costly than those of alkaline electrolyzers [21,30]. A schematic of the PEM electrolysis process is shown in Figure 6. 

The oxygen evolution reaction (OER) starts when hydrogen ions move to the cathode via the PEM due to an electrical potential applied at both electrodes releasing half a molecule of oxygen (O_2_) and electrons via the water splitting process. The hydrogen evolution reaction (HER) starts when hydrogen ions are reduced at the cathode, liberating one hydrogen (H_2_) molecule. 

The half-cell reaction at the anode in PEM water electrolysis is shown in Equation (4):

Anode: H_2_O → 2H^+^ + ½ O_2_ + 2e^−^ (4)

The half-cell reaction at the cathode in PEM water electrolysis is shown in Equation (5).

Cathode: 2H^+^ + 2e^−^ →H_2_ (5)

The overall reaction for PEM electrolysis is represented by Equation (3):

Overall: 2H_2_O → H_2_ + ½ O_2_ (6)

**Figure 7:**
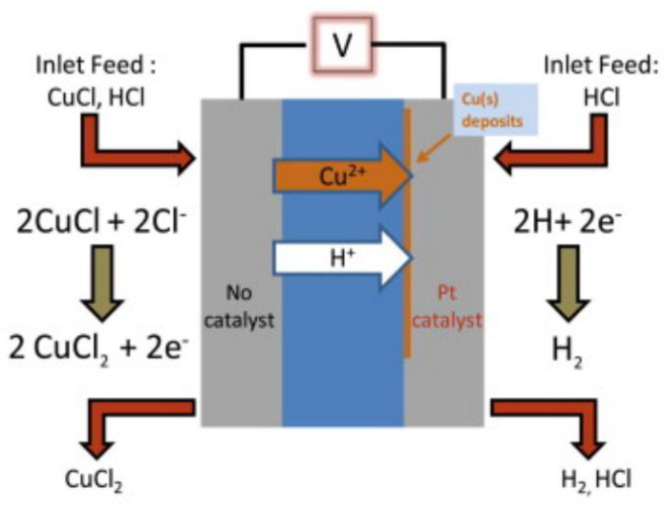
CuCl-HCl electrolysis schematic and chemical reactions. Reproduced with permission from [92].

Apart from traditional PEM water electrolysis, another type exists which utilizes copper chloride-hydrochloric acid (CuCl-HCl) as the electrolytes. In the past decade, studies carried out on CuCl-HCl electrolysis at low operating temperature (<80 °C) using a Nafion™ and Nafion™-based membranes revealed promising hydrogen production results [91,92,93,94,95,96,97]. A schematic of the CuCl-HCl electrolysis process is shown in Figure 7. The reaction for CuCl electrolysis produces two CuCl_2_ molecules and one H_.2_ molecule.

One study revealed that Nafion™ functions as an exceptional intermediate for ionic transfer without material compatibility issues in CuCl-HCl electrolytic systems when 0.2–1 M CuCl is added to the 2–10 M HCl electrolytes [92]. In some studies, milder electrolytes were utilized, with CuCl concentrations ranging from 0.01 to 0.2 M and HCl concentrations of 0.5–1 M [21,48]. However, some issues have been reported with Nafion™ membranes, namely, high copper diffusion, swelling, and the need for expensive membranes [97,98]. In contrast, a polybenzimidazole (PBI) membrane doped with phosphoric acid offers superior thermochemical and mechanical stabilities for working temperatures over 80 °C [21,98]. 

The half-cell reaction at the anode in CuCl-HCl electrolysis is shown in Equation (7):

Anode: 2CuCl + 2Cl^−^ → 2CuCl_2_ + 2e^−^ (7)

The half-cell reaction at the cathode in CuCl-HCl electrolysis is depicted in Equation (8).

Cathode: 2H^+^ + 2e^−^ → H_2_ (8)

The overall reaction for an alkaline electrolysis is represented by Equation (9):

Overall: 2CuCl + 2Cl^−^ + 2H^+^ → 2CuCl_2_ + H_2_ (9)

Recent findings suggested that high-temperature CuCl-HCl electrolysis using hybrid PBI/zirconium phosphate (PBI/ZrP) can increase the hydrogen production; therefore, this approach has the potential to make Nafion™ membranes redundant [21,48,98]. Kamaroddin et al. (2020) reported high-temperature CuCl-HCl electrolysis for hydrogen production at a temperature range of 100–130 °C with lower HCl concentration and electrolyte flowrate using a hybrid PBI/ZrP membrane [21]. Theirs was the first study that used a non-Nafion™ membrane in high-temperature CuCl-HCl electrolysis for hydrogen production. In another study, manipulations of the electrolyte concentrations and current densities were shown to increase hydrogen production, although this requires further investigation [48]. A summary of a CuCl-HCl electrolysis system for hydrogen production via Nafion™ and hybrid PBI/ZrP membrane is depicted in Table 1.

Choosing the right process using CuCl_2_ with spent residues can increase the yield of the electrolysis process [21,101]. CuCl_2_ can be further recycled to generate CuCl for the next round of electrolysis. Despite the challenges, hydrogen production via high-temperature electrolysis of CuCl-HCl can be seen as a suitable option because hydrogen is a clean, energy dense substance and a nontoxic energy source [48,102]. Therefore, high-temperature electrolysis of CuCl-HCl is a potential alternative to fossil fuels which may reduce the production costs of hydrogen by utilizing a cheaper membrane.

#### 3.2.2. Anion Exchange Membrane (AEM) Electrolysis

**Figure 8:**
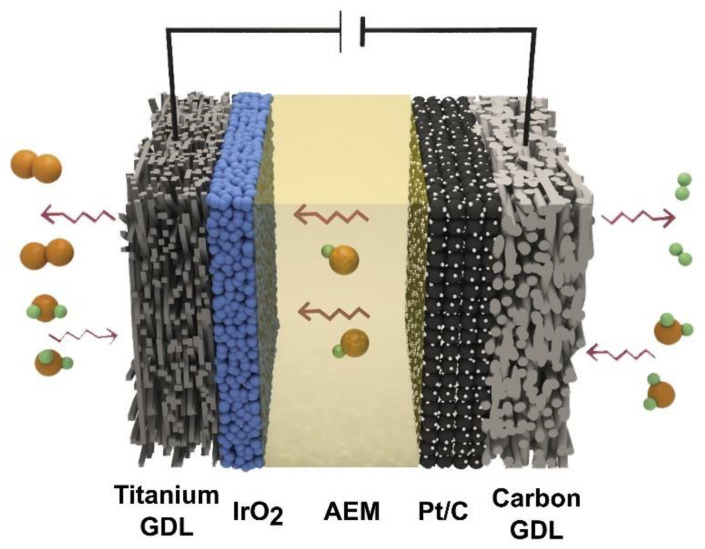
Single-cell components in AEM water electrolysis. Reproduced with permission from [107].

AEM water electrolysis is a hybrid method that combines the advantages of having PEM and alkaline electrolysis in a cell made up of a hydrocarbon-based anion exchange membrane and two transition metal (e.g., iridium (Ir), platinum (Pt), etc.) catalyst-based electrodes [71,81,103]. The advantages of this process compared to alkaline electrolysis are the use milder alkaline electrolytes or distilled water instead of a concentrated KOH solution and the possibilities of using a cheaper catalyst and an inexpensive nickel-based stack components [81,104]. However, current AEM electrolyzers shows low ionic conductivity, low power efficiency, medium range membrane stability with large Ohmic resistance loss and significant catalyst loading [81,105]. There is growing interest among the scientific community in developing a solid polymer anion exchange membrane, but more efforts are required regarding the catalyst design and synthesis [103,106]. A schematic and the overall cell reaction for AEM electrolysis are shown in Figure 8.

The half-cell reaction at the anode in an AEM electrolysis is shown in Equation (10):

Anode: 4OH^−^ → 2H_2_O + O_2_ + 4e^−^ (10)

The half-cell reaction at the cathode is depicted in Equation (11).

Cathode: 4H_2_O + 4e^−^ → 4OH^−^ + 2H_2_ (11)

The overall reaction for an AEM electrolysis is represented by Equation (12):

Overall: 4H_2_O → 2H_2_O + O_2_ + 2H_2_ (12)

A summary of the AEM electrolysis system is shown in Table 2. 

The above summary provides an overview of the type of membrane electrode assemblies, temperatures, membrane, types of electrolyte and voltages of the system. Common membranes used for AEM electrolysis are A-201, Takuyama, and Selemion AMV; a voltage range of 1.8–2.0 V is sufficient to produce hydrogen in the AEM electrolysis process. Currently, research on AEM is still at the laboratory scale, but recent studies have yielded significant information regarding the AEM electrolysis mechanism, as well as improvements of the electrocatalysts, membranes, electrodes, and membrane electrode assemblies (MEA) [27,81].

#### 3.2.3. Solid Oxide Electrolysis

**Figure 9:**
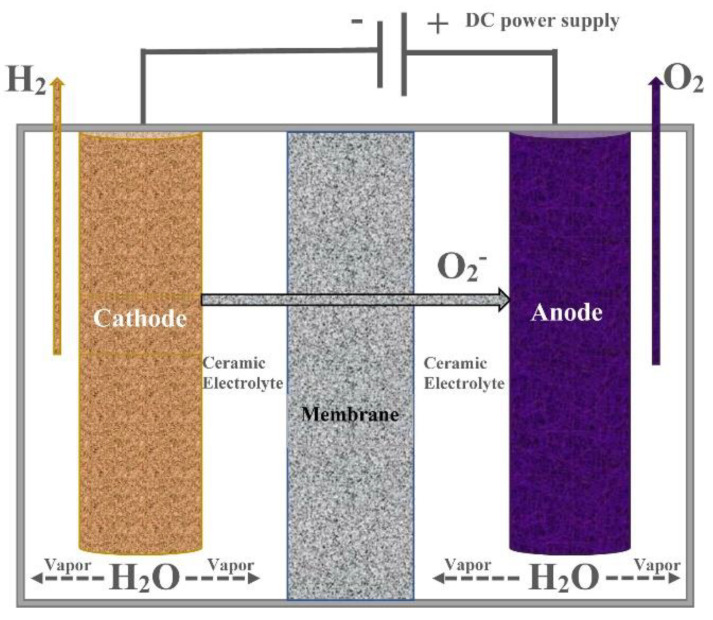
Solid oxide electrolysis and chemical species. Reproduced with permission from [114].

Solid oxide electrolysis (SOE) has received a lot of attention, as it is regarded as a high-efficiency process that converts electrical energy into chemical energy and produces high purity hydrogen [114]. Donitz and Erdle invented the technique in 1980, although it is still undergoing refinement [114]. SOE works at a high temperatures, i.e., 500–1000 °C, or the same as the output temperature of a nuclear reactor. SOE uses a solid ceramic membrane, which makes it compact and gives it a fast response time, i.e., comparable to that of a PEM electrolyzer cell. The advantages of having a solid-oxide electrolyzer include the fact that it can be a dual-function fuel cell/electrolyzer, and its superior ionic conductivity [29]. However, solid oxide electrolysis comes with a few disadvantages, e.g., the relative immaturity of the technology, the energy intensive nature of the process, high cost, low durability, and the need for ultrahigh operating temperatures [44,100]. Solid oxide electrolyzers (Figure 9) are unique on account of their need for high temperature operation, as extra heat input is required in addition to electrical input [29,114,115]. 

Half-cell reaction at the anode in solid oxide electrolysis is shown in Equation (13):

Anode: O^2−^ → ½ O_2_ + 2e^−^ (13)

Half-cell reaction at the cathode is depicted in Equation (14).

Cathode: H_2_O + 2e^−^ → H_2_ + O^2-^ (14)

The overall reaction for a solid oxide electrolysis is represented by Equation (15):

Overall: H_2_O → H_2_ + ½ O_2_ (15)

A summary of a SOE system for membrane-based electrolysis is shown in Table 3. SOE operating temperatures and voltages range from 700 to 800 °C and 0.95 to 1.40 V, respectively. The majority of the SOE systems use water as the electrolysis reactant. 

SOE holds great promise if we are able to utilize the waste heat from power plants or other chemical processes as heat sources.

#### 3.2.4. Microbial Electrolysis

**Figure 10:**
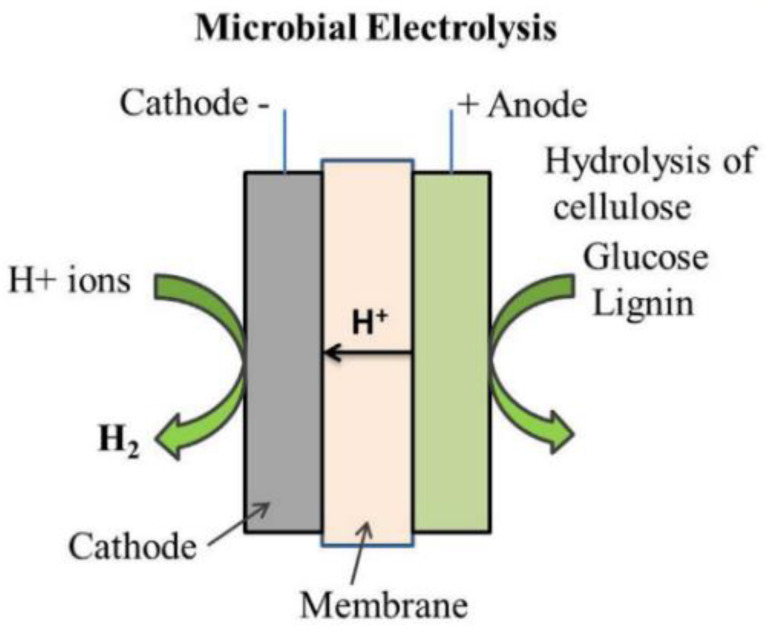
Microbial electrolysis. Reproduced with permission from [19].

Microbial electrolysis cell (MEC) technology is capable of producing hydrogen from organic matter, including wastewaters and industrial biomass waste. In MECs, electrical energy is transformed into chemical energy. MEC technology is very similar to that of microbial fuel cells (MFCs), except that the operating concept is the opposite [121]. In 2005, two university groups from Penn State University, USA, and Wageningen University in the Netherlands, presented the first microbial electrolysis cell (MEC) method [20,122]. A schematic for microbial electrolysis is shown in Figure 10.

The half-cell reaction at the anode in a microbial electrolysis is shown in Equation (16):

Anode: CH_3_COO^−^ + 4H_2_O → 2HCO^3−^ + 9H^+^ + 8e^−^ (16)

The half-cell reaction at the cathode is depicted in Equation (17).

Cathode: 8H^+^ + 8e^−^ →4H_2_ (17)

The overall reaction for a microbial electrolysis is represented by Equation (18):

Overall: CH_3_COO^−^ + 4H_2_O → 2HCO^3−^ + H^+^ + 4H_2_ (18)

The evolution of microbial electrolysis cell technology from 2005 to 2021 is summarized in Table 4. In previous studies, several membranes, e.g., SPEEK, SPEEK/PES, SPAES/polyimide, SPEEK/PES, Nafion™, AMI-7001, bipolar membranes, charge-mosaic membranes, and microporous membranes, were tested and showed promising results in microbial electrolysis cells [123,124,125,126]. The advantages of MEC include the fact that it can generate hydrogen from organic molecules under the influence of a low external voltage [126,127]. However, there are disadvantages that need to be taken into account, e.g., it has high internal resistance, a complicated design, high fabrication and operation costs, and is a technology that is still under development [128].

#### 3.2.5. Acid-Alkaline Amphoteric Electrolysis

**Figure 11:**
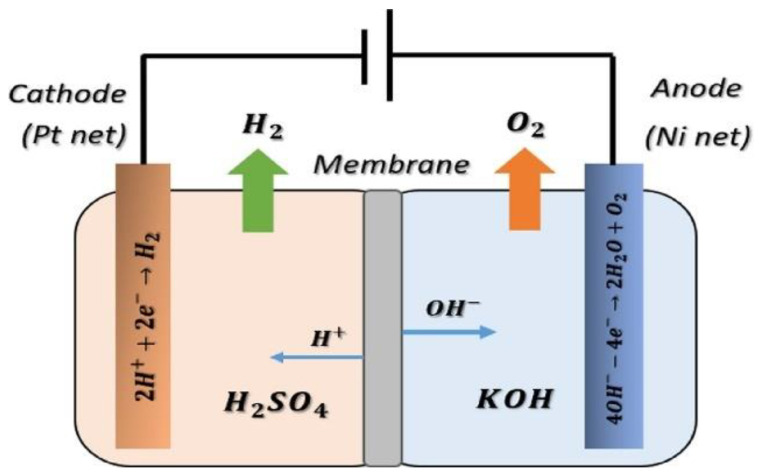
Acidic-alkaline amphoteric electrolysis. Reproduced with permission from [34].

Large-scale, acid-alkaline amphoteric (AAA) water electrolysis is deemed a promising method for effective hydrogen generation; however, a functionalized polymer for constructing membranes is still either unable to yield good electrolysis performance or is not durable enough [34,83]. Current studies on a variety of H_2_SO_4_-doped PBI-based membranes for use in AAA water electrolysis systems (Figure 11), including poly (2,2’(m-phenylene)-5,5’-bibenzimidazole) (m-PBI), poly (4,4’diphenylether-5,5’-bibenzimidazole) (OPBI), Nafion™ 117 (N117) and Nafion™ 115 (N115), are considered to be good potential membranes for amphoteric electrolysis [83,139].

The half-cell reaction at the anode in acid-alkaline amphoteric electrolysis is shown in Equation (19):

Anode: 4OH^−^ → 2H_2_O + O_2_ + 4e^−^ (19)

The half-cell reaction at the cathode is depicted in Equation (20).

Cathode: 2H^+^ + 2e^−^ → H_2_ (20)

The overall reaction for an acid-alkaline amphoteric electrolysis is represented by Equation (21):

Overall: 4OH^−^ + 4H^+^ → 2H_2_O + O_2_ + 2H_2_ (21)

The summary of acid-alkaline amphoteric electrolysis for hydrogen production is depicted in Table 5. The electrolytes used are sulfuric acid (H_2_SO_4_) and potassium hydroxide (KOH) with operating conditions range from 20 to 60 °C (temperature), 1.98 to 2.2 V (voltage) and 200 to 800 A cm^−2^ (current density). It was reported that the AAA electrolysis efficiency can consistently achieved up to 100% with few more advantages which includes a reduction in overpotential and energy consumption (30% of pure alkaline electrolysis requirement) and up to 4 times more hydrogen production compared to alkaline electrolysis [34]. However, due to characteristic of the AAA, the setup has higher membrane resistance compare to alkaline electrolysis, the need to use bipolar ion-exchange membrane and it requires simultaneous usage of acidic and alkaline electrolytes in the system [83].

#### 3.2.6. Photoelectrochemical Electrolysis

**Figure 12:**
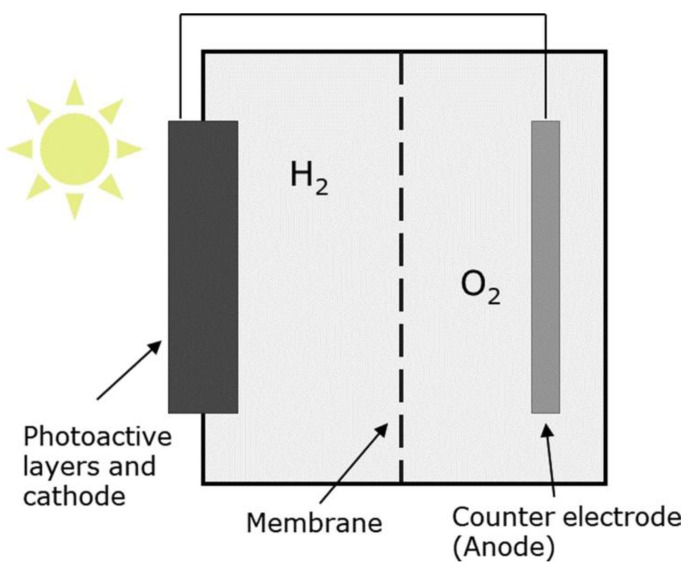
Photoelectrochemical electrolysis. Reproduced with permission from [142].

The first report of photoelectrochemical (PEC) water splitting was published in 1970s, when a conductive electrode composed of TiO_2_ was illuminated in aqueous solution [140,141]. Photoelectrochemical (PEC) electrolysis system (Figure 12), which converts solar energy directly to hydrogen by using a direct and simple setup has sparked considerable attention in recent years. Water decomposes into hydrogen and oxygen by absorbing solar photons in a semiconductor material attached with electrocatalysts [142,143,144].

In the PEC approach, photocatalysts are first produced as electrodes on conductive substrates, and a modest bias is then applied for water splitting. The half-cell reaction at the anode in PEC electrolysis is shown in Equation (22):

Anode: 2H_2_O → 4H^+^ + O_2_ + 4e^−^ (22)

The half-cell reaction at the cathode is depicted in Equation (23).

Cathode: 4H^+^ + 4e^−^ →2H_2_ (23)

The overall reaction for a PEC electrolysis is represented by Equation (24):

Overall: 2H_2_O + 4H^+^ → 4H^+^ + O_2_ + 2H_2_ (24)

Table 6 shows a summary of photoelectrochemical electrolysis for hydrogen production in which the membrane is integrated with a semiconductor material in order to activate the photoelectrochemical reaction when immersed in an appropriate agent (e.g., methanol, water or ethanol). PEC electrolysis, that can directly use the free energy obtained from solar panels to produce hydrogen, holds tremendous potential due to its simple setup, although efficiency remains quite low < 10% due to the fact that the technology is still in its infancy [142,144]. Therefore, there should be collaborative efforts in the scientific community to increase the efficiency of PEC electrolysis, with the goal of matching that of photovoltaic assisted water splitting processes.

### 3.3. Summary

Various methods of hydrogen production have been discussed above, each of which has its advantages and disadvantages. At the end of the day, we need to consider the greenest possible way to generate hydrogen without releasing greenhouse gases into the atmosphere in an effort to curb climate change. The efficiency of the electrolyzer for hydrogen production can be determined using the formulae in Equations (25)–(27):

Efficiency (%) = V_H2 real_/V_H2 ideal_ × 100 (25)

with V_H2 real_ = V_H2 measured_ × T_standard_/T_measured_ and; (26)

V_H2 ideal_ = I × V_m_ × t/(2 × F) (27)

where I is current (A), V_m_ is a molar volume of an ideal gas, t is time (s) and F is Faraday’s constant, i.e., 96485 A.s/mol.

A summary of hydrogen production by water splitting technologies, along with their advantages, disadvantages and efficiencies, is depicted in Table 7.

**Figure 13:**
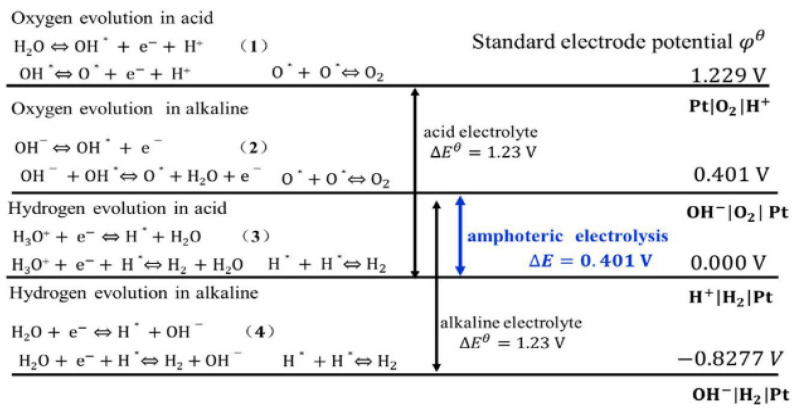
Standard potential for water splitting under different pH conditions [34].

Since water splitting technologies mainly involve either oxygen evolution (OER) or hydrogen evolution (HER) reactions at their respective electrodes, the electrode potentials can be summarized for acidic and alkaline media. A summary of electrode half potentials for various membrane-based electrolysis for hydrogen production is given in Figure 13. 

This is a comprehensive way of visualizing the electrode half potentials for various membrane-based electrolysis systems, as there are duplications for the anode and cathode half-cell reactions. Despite using a different type of membrane, the process is nonetheless based on the water splitting technologies, and the standard electrode potential is the same for both acid and alkaline electrolysis (1.23 V), except with amphoteric electrolysis, which has only 0.401 V.

It can be concluded that each water splitting technology has its advantages and disadvantages. However, membrane-based electrolysis appears to offer a lot of potential for hydrogen production. Therefore, the research, development and commercialization of more economical membranes should be a major focus if we are to exploit the full potential of these technologies.

## 4. Parameters Affecting the Membrane-Based Electrolysis 

Many factors determine the electrolyzer performance in an electrolysis system [14]. Apart from the selection of an appropriate material for the construction of the electrolyzer, the operating parameters affecting hydrogen yield are very important [14,152,153]. In this study, four operating parameters influencing hydrogen production in membrane-based electrolysis are outlined, i.e., temperature, electrolysis concentration, electrolysis flowrate and miscellaneous.

### 4.1. Temperature

Alkaline electrolysis is the most established hydrogen production technology; it is generally applied for industrial-scale electrolytic hydrogen production with a typical operating temperature of 40–90 °C [114], or 30–100 °C if highly concentrated KOH is used, with an estimated overall efficiency of 70–80% [154]. On the other hand, a typical PEM electrolysis process operates at between 30–90 °C, with a standard Nafion™-based membrane being the core component of the membrane electrode [88,155,156]. Although some studies have reported the use of PEM electrolyzers at high temperature, efforts in this endeavor were hindered by the inability of the Nafion™ membrane to withstand operating temperatures above 90 °C, as this leads to mechanical degradation and a loss of ionic conductivity [44,157,158]. Toghyani et. al. (2018) reported that the hydrogen and oxygen reaction rate increased dramatically at higher operating temperatures as a result of the faster kinetics of the electrochemical reactions [63]. Recently, Kamaroddin et al. (2020) revealed a PBI/ZrP hybrid membrane that can operate at 100–130 °C by synthesizing a PBI-based hybrid membrane using a solution mixing method with the addition of a ZrO_2_ inorganic filler, followed by phosphoric acid doping. Therefore, by better integrating the polymer backbone through the use of ZrO_2,_ more acid sites attach to the PBI, resulting in enhanced proton movement via the Grotthus mechanism, as well as improved ionic conductivity, tensile strength and ion exchange capacity [21].

The operating temperature required for water splitting technologies for hydrogen production is often noted as one of the biggest factors influencing operation costs. SOE requires the highest operating temperature, i.e., 500–1000 °C, but has an efficiency close to 100% [25,159]. Due to its advantageous thermodynamics and kinetics, high temperature steam water electrolysis can deliver high efficiency at a lower overall cost than conventional low temperature electrolysis [114,156]. Furthermore, because of the operating temperatures of SOEs, it is possible to simultaneously electrolyze CO_2_ and H_2_O. However, in order for a system to be feasible, the heat must be from a renewable source, or from exothermic waste heat [31,64,160].

### 4.2. Electrolytes Concentration

In some water splitting processes, electrolyte concentration plays a vital role in determining the rate of the reaction and the amount of the hydrogen produced [44,159]. Chakik et al. (2017) reported that the amount of hydrogen produced is strongly correlated with the electrolyte concentration, i.e., a higher concentration in the electrolyte increases the ionic conductivity of the solution which, in turn, promotes hydrogen evolution reactions and improves yield [161]. According to Lei et al. (2019), a quadruple increase was observed in the hydrogen production rate in an amphoteric electrolysis that used 4 M KOH and 2 M H_2_SO_4_ within a temperature range of 30 to 50 °C. 

However, excessive electrolyte concentration can deteriorate the MEA components including the membrane, electrodes, gasket, current collector, bipolar plates, etc., which, in turn, affects the hydrogen yield [47,162,163]. The corrosive nature of concentrated electrolytes, be it acidic or alkaline, can also cause damage to the peristaltic pump and its components, the electrolyte tubing, thermocouples, heating elements, etc. [27,164]. Therefore, considerable research must be carried out regarding a suitable electrolyte concentration to achieve the optimum concentration to maximize yield.

### 4.3. Electrolytes Flowrate

As the membrane serves as the core component in the electrolyzer, the flowrate of the electrolytes is a crucial factor that determines the kinetics of the electrolysis reaction [21]. Although many elements of the electrolyzer have improved over the previous decade, the impacts of the various operating parameters are still being investigated. Notably, the main limiting variables have yet to be identified using kinetic and thermodynamic relationships [165,166]. However, a faster electrolyte flowrate will not necessarily increase the rate of hydrogen production, and instead may be a limiting factor, negatively influencing the rate of ionization in an HER or OER. Therefore, a good balance between an optimal electrolyte flowrate and other parameters must be found in order to achieve an optimal hydrogen yield, depending on the type of available energy. 

### 4.4. Others

Apart from all the above parameters, the electrode material plays an important role in the electrolyzer setup in terms of ensuring a durable and highly efficient process. The electrode materials should be nonreactive with excellent corrosion resistance, good proton conductivity, and the ability to support active catalytic activity for HER and OER [154,167]. 

The volume of hydrogen generated during the electrolysis process grows steadily as the applied current increases [161,168]. Moreover, both the catalyst composition and its morphology function as synergistic factors that enhance HER and OER [20]. As a rule of thumb, the MEA manufacturing procedure is critical in defining performance, production costs, and durability [169,170].

## 5. Challenges and Future Trends 

The present overview of membrane-based electrolysis approaches for hydrogen production is largely based upon the results and discussions presented in the literature. A literature search in the Web of Science Core Collection portal (accessed 1 September, 2021) for alkaline, solid oxide, PEM, AEM, acidic-alkaline amphoteric, microbial, and PEC membrane-based electrolysis yielded a total of 1193 results from over a 10-year time span (2010–2021) using as keywords “membrane electrolysis” and “hydrogen production”. For a comparison, a quick search on the MDPI portal yielded only 20 results for this period using the same keywords.

The past decade has witnessed growing research interest in membrane-based electrolysis for hydrogen production. This is due to the increasing demand for green energy and the implementation of zero carbon footprint initiatives. Nevertheless, there are still major obstacles which need to be overcome before membrane-based electrolysis can be considered an economically viable, large-scale hydrogen generation solution, including the cost, availability, and the durability of the membrane, type of catalyst, the cost of using platinum group metal-based catalysts, and corrosion problems associated with the electrodes and separator plates. 

## 6. Conclusions

Despite significant advancements in the development of all required components for membrane-based electrolytic hydrogen production systems, giving rise to significant improvements in durability, performance and efficiency, some electrolysis technologies are still in the early stages of development, e.g., solid-oxide, anion exchange membrane, microbial and photoelectrochemical electrolysis. In this review, we have provided a short introduction to various water splitting technologies for hydrogen production, including discussion of the type of membrane that are currently being used and the associated progress in their development. In addition, we have highlighted and emphasized recent development in membrane-based electrolysis. The present review not only discusses in detail the availability of the hydrogen production technology, but also summarizes trends of PEM water splitting technologies over the past decade, presenting a review of hydrogen production including the advantages, disadvantages and efficiencies of the various technologies. Parameters affecting the performance of membrane-based electrolysis are also discussed. Finally, we have summarized the challenges to the development of membrane-based electrolysis technologies, and have outlined our ideas for future research directions with the aim of fully tapping into this potential energy source which has a zero-carbon footprint. Future development of membranes for water splitting technologies, especially for the membrane-based electrolysis, should be focused on more economical models, like PBI, SPEEK, polysulfone, polyimides, polyethylene etc., in order to fully benefit from the emerging hydrogen economy ecosystem via the creation of efficient hydrogen generators for fuel cell cars and fuel cell power supply, as well as mobile electrolyzers to power critical equipment in remote areas such as telecommunication towers or safety and security surveillance. The application of membrane-based electrolysis and other auxiliary equipment allowing the use of hydrogen in transportation and industrial activities will be of at great interest over the next 5 to 10 years.

## Abbreviation

AEMAnion exchange membraneAAAAcidic-alkaline amphotericPAPhosphoric acidPBIPolybenzimidazolePEEKPoly ether ether ketonePEMProton Exchange MembraneSPEEKSulfonated poly ether ether ketoneMECMicrobial electrolysis cellPECPhotoelectrochemicalSOESolid oxide electrolysisCuCl-HClCopper chloride-hydrochloric acidOEROxygen evolution reactionHERHydrogen evolution reactionMFCMicrobial fuel cellGHGGreenhouse gasesPBI/ZrPPolybenzimidazole/Zirconium phosphatePEMEProton exchange membrane electrolyzerPFSAPerfluorinated sulfonic acidPEMFCProton exchange membrane fuel cellMEAMembrane electrode assembliesSPESSulfonated polyether sulfoneYSZYittria stabilized zirconiaCGOGadolinium doped ceriaSSZScadinia stabilized zirconiaLDCLanthanum doped ceriumLSGMLanthanum gallate-based electrolyteSPAESSulfonated Polyaryl Ether Sulfone

## Figures and Tables

**Table 1:** CuCl-HCl electrolysis system for hydrogen production via hybrid PBI/ZrP and Nafion™ membrane.

Authors	Electrolyte(s) Concentration (M)	Temperature (°C)	Membrane	Electrolyte Flowrate (cm^3^ min^−1^)
Kamaroddin M.F.A et al., 2020 [21]	0.01–0.2 M CuCl	100–130	PBI/ZrP	CuCl: 3–30 HCl: 3–30
	1 M HCl			
Abdo & Easton 2016 [95]	0.2 M CuCl, 2 M HCl	25	Nafion™/Polyaniline (PANI)	CuCl/HCl: 60 DI water: 60
	DI water			
Naterer et al., 2015 [94]	0.5–1.0 M CuCl	45–60	Nafion™ 117	CuCl: 600
	6–10 M HCl		HYDRion	HCl: 600
Aghahosseini et al., 2013 [99]	0.5–1.0 M CuCl	25–60	Nafion™ 117	CuCl: 100–500
	6–10 M HCl			HCl: 100–500
Edge 2013 [91]	0.002–0.2 M CuCl	25–80	Nafion™	CuCl: 40–200
	2 M HCl			HCl: 40–200
Schatz et al., 2013 [100]	1–2 M CuCl	80	Nafion™	CuCl: 59
	6 M HCl			HCl: 130
Balashov 2011 [92]	0.2–1.0 M CuCl	22–30	Nafion™ 115	CuCl: 30 & 68
	2 M HCl			HCl: 28.5
Gong et al., 2010 [100]	0.2–1.0 M CuCl	24–65	Nafion™	CuCl: 3.4–22
	2–6 M HCl			HCl: 4.4–27

**Table 2:** Summary of the AEM electrolysis system for hydrogen production.

Authors	Membrane Electrode Assembly GDL * (anode/cathode)	Temperature (°C)	Membrane	Electrolyte	Voltage (V)
Leng et al., 2012 [108]	Ti foam/Ti foam	50	A-201, Takuyama	Deionized water	1.8
Pavel et al., 2014 [109]	Ni foam/carbon cloth	50	A-201 Takuyama	1% K_2_CO_3_/KHCO_3_	1.9
Xiao et al., 2012 [110]	Ni form/stainless steel fiber felt	70	xQAPS	Ultrapure water	1.85
Wu et al., 2011 [111]	Stainless steel mesh/stainless steel mesh	25	Quaternary ammonium	1 M KOH	1.8
Seetharaman et al., 2013 [112]	NiO/NiO	80	Selemion AMV	0–5.36 M KOH	1.9
Joe et al., 2014 [113]	Ni oxide/Ni	30	Selemion AMV	Deionized water	2.0

* GDL—gas diffusion layer.

**Table 3:** Summary of SOE system for hydrogen production.

References	Membrane	Temperature (°C)	Durability Test Time (h)	Electrolysis Reactant	Voltage (V)
[116]	YSZ */CGO	750	120	H_2_O	1.15
[117]	SSZ	700	330	H_2_O	1.30
[118]	SSZ	700	1000	H_2_O	1.30
[119]	LDC/LSGM/LDC	800	-	H_2_O	0.95
[120]	YSZ *	800	300	H_2_O/CO_2_	1.40

* YSZ—Yittria-zirconized zirconia, CGO—Gadolinium doped ceria, SSZ—Scadinia stabilized. zirconia, LDC—lanthanum doped cerium, LSGM—La0.9Sr0.1Ga0.8Mg0.2O3−δ.

**Table 4:** Chronological development of microbial electrolysis cell (MEC) technology from 2005 to 2021.

Year	Description	References
2005	Hydrogen gas generated from acetate using a full anaerobic microbial fuel cell	[129]
2008	Biocathode was used in MEC	[130]
2009	Effort to increase the hydrogen production by using an economical cathode SS A286 and nickel	[131]
2010	Establishment of a life cycle assessment for microbial electrolysis cells	[132]
2012	Conversion of CO_2_ to methane using MEC technology	[133]
2015	Dark fermentation and MEC were integrated and evaluated by producing hydrogen from sugar beet juice	[134]
2016	Removal of cadmium by using MEC	[135]
2018	Prefermentation of MEC as the medium with which to check the role of free nitrous acid	[136]
2019	A method to quantify the internal resistance of MECs was developed	[137]
2020	The effectiveness of chloroform as a homoacetogen inhibitor was demonstrated	[138]
2021	The effect of high applied voltages on bioanodes in the presence of chlorides was studied	[81]

**Table 5:** Summary of acid-alkaline amphoteric electrolysis for hydrogen production.

References	Electrolyte(s) Concentration (M)	Temperature (°C)	Membrane	Voltage (V)	Current Density (A cm^−2^)
[138]	3 M H_2_SO_4_/6 M KOH	20	PBI/Graphitic carbon nitride	1.98	800
[83]	1–3 M H_2_SO_4_/6 M KOH	20–60	Nafion 115,	2.0	800
			OPBI, m-PBI		
[34]	1–2 M H_2_SO_4_/2–4 KOH	30–50	Nafion 115	2.2	200

**Table 6:** Summary of photoelectrochemical electrolysis for hydrogen production.

References	Membrane	Agent	Reactor
[145]	TiO_2_-Nafion-Pt	Methanol	-
[146]	Pt/SrTiO_3_Rh-Nafion	Water	H-type integrated
[147]	BiVO_4_-Nafion	Water	Dual
[148]	Porous Nafion-Pt-TiO_2_	Ethanol	
[149]	WO_3_-TiO_2_-Pt-Nafion	Water	H-type
[150]	Carbon coated Degussa TiO_2_-P25	Water	-
[151]	Nafion, FKE Fumatech, sulfonated polyethersulfone (sPES), sPES/mesoporous-Si-MCM41-nanoparticles	Water	-

**Table 7:** Summary of hydrogen production by water splitting technologies along with the types of diaphragm/membrane used, advantages, disadvantages, and efficiencies [19,21,27,34,81,83,114,128,142].

Water Splitting Technologies	Advantages	Disadvantages	Efficiency
Alkaline Type of diaphragm: porous inorganic (asbestos, ceramic, cement)	Well established technology Economical Very durable Operates at low temperature (30–80 °C) Inexpensive electrocatalyst	High concentration corrosive electrolytes Limited current density (below 400 mA/cm^2^) Low operating pressure Low energy efficiency Low gas purity	60–80%
Solid oxide Types of membranes: oxygen ion ceramic electrolyte membrane, YSZ	Dual-function fuel cell and electrolyzer Superior ionic conductivity Ultrapure hydrogen Excellent efficiency	Very high operating temperature (500–850 °C) Energy intensive process and not economical Low durability (stability and degradation)Still immature technology—lab scale	90–~100%
PEM Type of membranes: Nafion™, PBI, SPEEK, polyethylene	High hydrogen purity (up to 99.995%), High current density High voltage efficiency Dynamic operation	High-cost catalysts Mildly durable Costly membrane More expensive stack materials compared to alkaline Partially established technology	70–90%
AEM Types of membranes: A201 membrane, Selenion AMV, A901 membrane	Lower cost of catalysts Inexpensive stack components -(Nickel-based)	Low ionic conductivity Early stage of development Low power efficiency Low membrane stability Large Ohmic resistance loss Large catalyst loading	50–70%
Acid-alkaline amphoteric Types of membranes: bipolar membrane, acid-doped PBI-based membranes, Nafion™	Reduced energy consumption Reduced overpotential Hydrogen production four times that of alkaline electrolysis	Increased membrane resistance Need to use bipolar ion-exchange membrane Need to use both acidic and alkaline electrolytes	~100%
Microbial Types of membranes: SPAES */polyimide, SPEEK, SPEEK/PES, Nafion™, AMI-7001, bipolar membranes, charge-mosaic membranes, microporous membranes	Requires only a low external voltage Uses organic materials	Still under development High internal resistance Complicated design Low rates of hydrogen production Fabrication and operational costs are high	60–70%
Photoelectrochemical Types of membranes: polyamide, Nafion™ based membrane	Direct solar to hydrogen conversion Simpler setup	Low conversion factor Low hydrogen production Still at infancy stage	<10%

* SPAES—sulfonated poly (arylene ether sulfone).

## Data Availability

Not applicable.
